# Effect of lower limb alignment on outcome after lateral unicompartmental knee arthroplasty: a retrospective study

**DOI:** 10.1186/s12891-024-07208-4

**Published:** 2024-01-20

**Authors:** Tong Zheng, Dehua Liu, Ziyue Chu, Yange Luo, Qunshan Lu, Baoqing Zhang, Peilai Liu

**Affiliations:** 1 Department of Orthopaedics, Qilu Hospital of Shandong University, Jinan, 250102 China; 2 Cheeloo College of Medicine, Shandong University, Jinan, 250102 China

**Keywords:** Lateral unicompartmental knee arthroplasty, Valgus, Lower limb alignment, Patients’ outcome

## Abstract

**Purpose:**

The objective of this study was to investigate the correlation between lower limb alignment and patient outcomes after lateral unicompartmental knee arthroplasty (LUKA).

**Methods:**

In this retrospective study, the information of 51 patients who underwent lateral UKA was collected after an average of 27months of follow-up (13 to 60 months). Evaluation indicators include the AKS and WOMAC score. The Kellgren-Lawrence grade is used to evaluate the severity of osteoarthritis, while the hip-knee-ankle (HKA) angle is utilized to measure the valgus angle of lower limb alignment.

**Result:**

Patients with postoperative valgus (≥ 3°) alignment had the best outcomes, while those with varus (≤-3°) alignment had the worst outcomes (*p* < 0.001). Furthermore, it was noted that patients with preoperative mild valgus (≤ 4°) alignment had worse postoperative outcomes than those with severe valgus (≥ 7°) alignment (*p* < 0.05). The study also revealed a positive correlation between postoperative valgus and WOMAC scores (*p* < 0.001), whereas a negative correlation was observed between the change in valgus angle and WOMAC scores (*p* = 0.005).

**Conclusion:**

During follow-ups, we found that lower limb alignment seems to be an independent predictor of postoperative outcomes. It is recommended that more than 3° of valgus alignment should be maintained after LUKA. Surgeons performing lateral UKA should be cautious of overcorrecting alignment, particularly in patients with preoperative mild valgus alignment.

**Supplementary Information:**

The online version contains supplementary material available at 10.1186/s12891-024-07208-4.

## Introduction

Osteoarthritis of the knee is a common kind of degenerative disease; approximately 85% of patients have degeneration limited to unilateral compartments [1], and 10% of patients have isolated lateral compartment disease [2].

Unicompartmental knee arthroplasty (UKA) has emerged as a dependable method for treating unicompartmental osteoarthritis. It offers more advantages than total knee arthroplasty (TKA), including faster recovery, improved kinematics, and better functional outcomes [3–5]. Many studies have shown that partial varus should remain after medial UKA [6], but there are few similar studies of lateral unicompartmental knee arthroplasty (LUKA) [7]. Due to the distinctive anatomy and kinematic mechanism of the lateral compartment of the knee [8, 9],the use of a fixed-bearing prosthesis is recommended for lateral UKA [10, 11].It should be noted that research conclusions pertaining to medial UKA cannot be directly applied to lateral UKA. Patients suffering from lateral compartment osteoarthritis commonly manifest knee valgus deformity [12]. Preoperative and postoperative lower limb alignment may have a significant impact on patient outcomes. However, there is a paucity of research on the correlation between preoperative and postoperative alignment and the differences in the clinical and functional performance of patients with varus, neutral, and valgus alignment.

This study retrospectively analysed the changes in lower limb alignment and the correlation between alignment and the clinical and functional scores ofpatients who underwent lateral UKA. The aim is to provide recommendations on the surgical indications, techniques, and goals. The hypothesis being tested is that mild valgus alignment post-surgery leads to better clinical and functional outcomes.

## Materials and methods

**Fig. 1:**
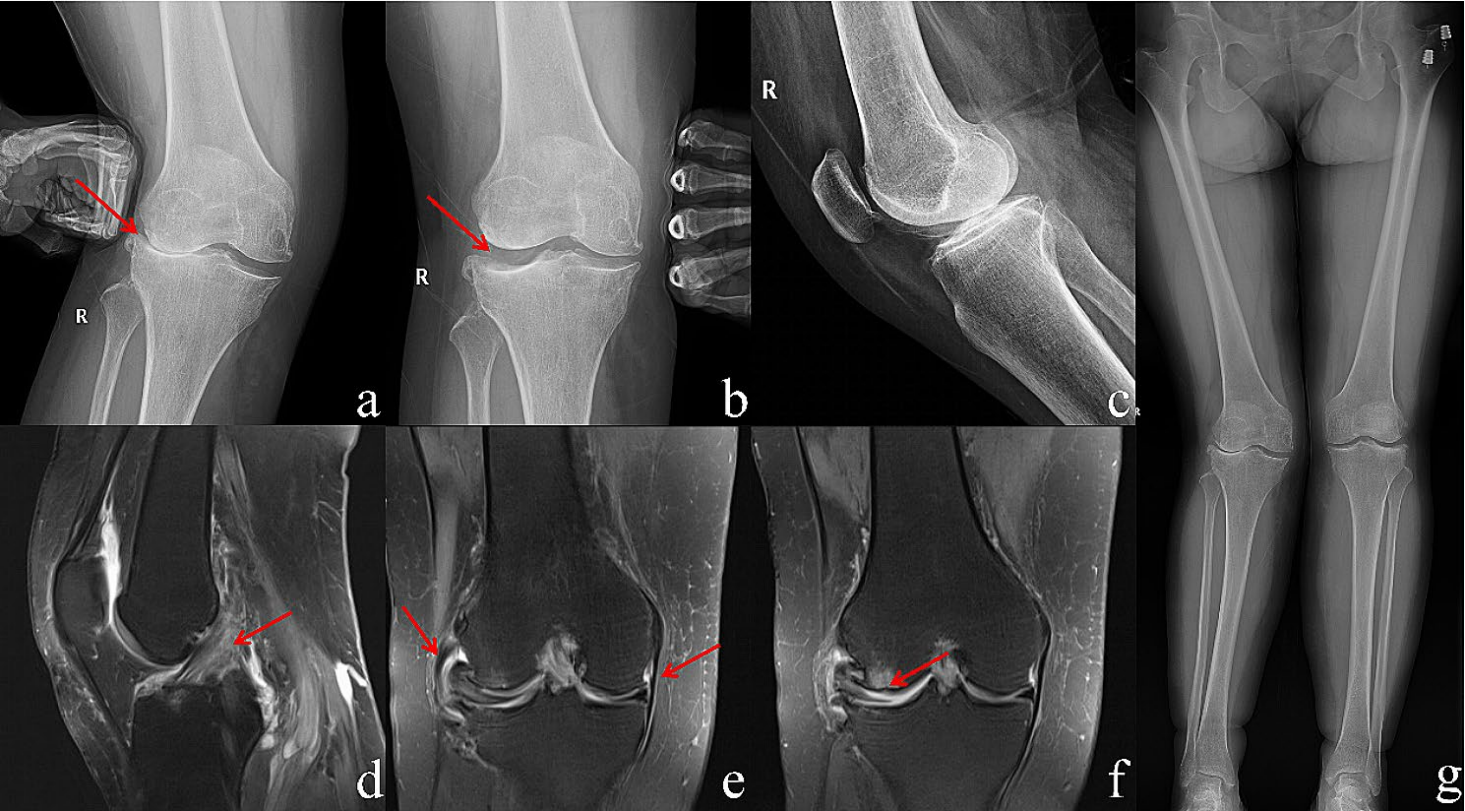
Preoperative imaging examination for LUKA Preoperative X-ray and knee MRI examinations are necessary to assess the suitability of the surgery. X-ray can initially identify simple lateral compartment osteoarthritis (**a**, **b**, **c**, and **g**) and determine if valgus correction is possible (**b**). MRI can confirm the condition of the ACL (**d**) and medial and lateral collateral ligaments (**e**) and verify the presence of worn cartilage only in the lateral compartment (**f**)

A total of 51 lateral UKAs (49 patients) were performed between August 2018 and August 2022, and the mean follow-up period was 27 months (ranging from 13 to 60 months). Of these patients, two underwent bilateral lateral UKAs at the same time. Surgical inclusion criteria included [1] isolated lateral compartment osteoarthritis; [2] intact cruciate and collateral ligaments; [3] knee flexion contracture < 10°; and [4] passively correctable knee valgus deformity [2] (Fig. 1).

In this study, all patients underwent fixed-bearing lateral UKAs (DePuy Sigma high-performance partial knee) using a standardized surgical protocol through the lateral parapatellar approach. The senior surgeon utilized the same technique for each procedure, achieving good flexion and extension gaps as well as tissue balance during surgery. The surgery was performed by the same professor.

**Fig. 2:**
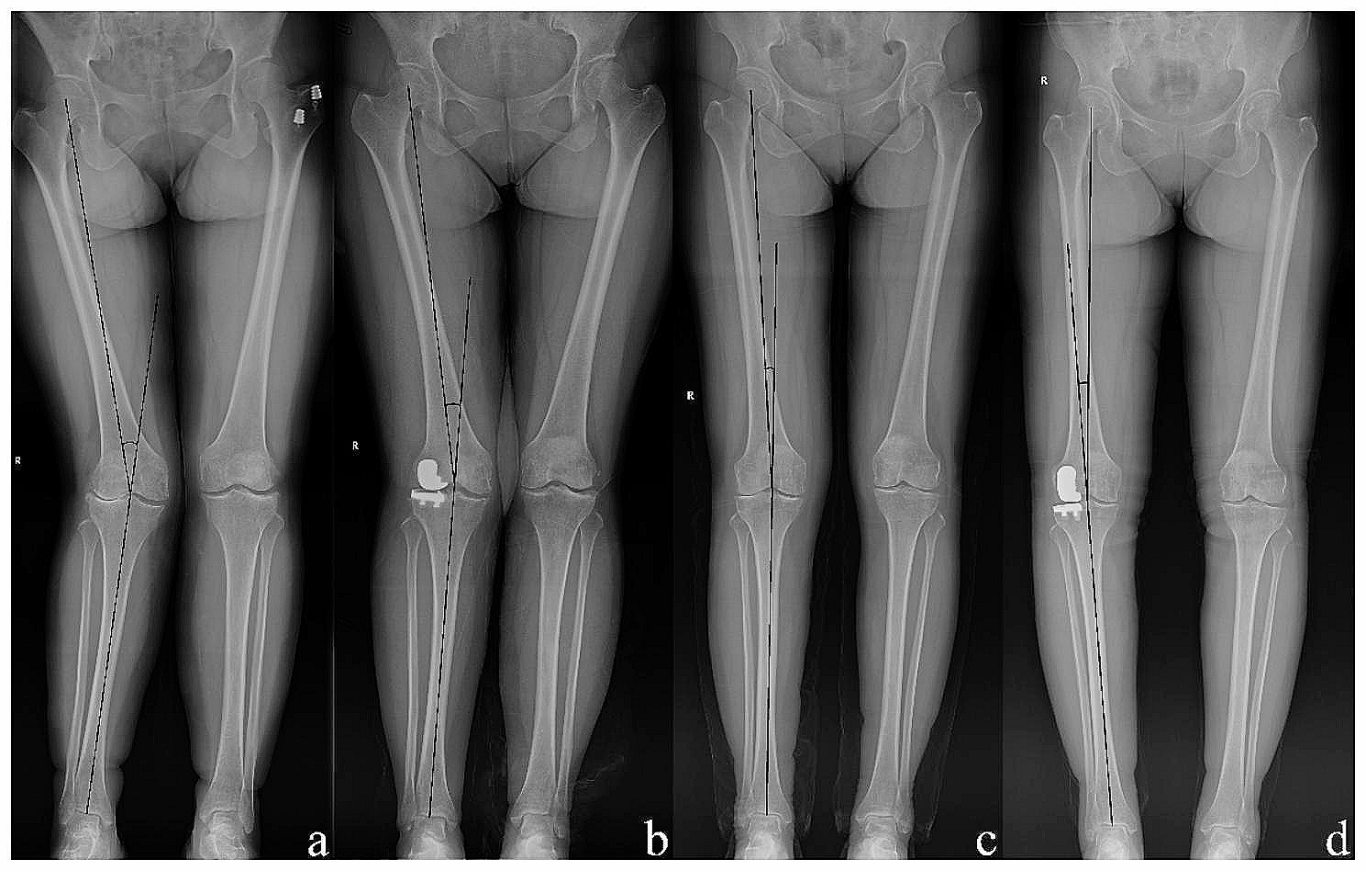
The measurement method of lower limb alignment and typical cases The femoral mechanical axis is determined by drawing a line connecting the centre of the femoral head and the midpoint of the femoral condyle. The tibial mechanical axis is determined by drawing a line connecting the midpoint of the tibial shaft (spinous process) and the midpoint of the talus. The HKA angle is the angle formed by the intersection of these two lines. In typical cases, the HKA angle changed from 16.61° preoperatively (**a**) to 10.15° postoperatively (**b**) and from 4.95° (**c**) to -4.50° (**d**)

We recorded basic patient characteristics, including age, sex, and body mass index (BMI) from the database. Clinical examinations and radiological examinations (anteroposterior and lateral X-rays of the knee joint) were performed, and the severity of knee osteoarthritis (KOA) was assessed using the Kellgren-Lawrence grade before the operation [13, 14]. Preoperative and 3-month postoperative full-length anteroposterior X-rays of the lower limbs (hip to ankle) were obtained to measure the hip-knee-ankle (HKA) angle, which is the angle formed by the mechanical axes of the tibia and femur, to analyse lower limb alignment [15–17]. Conventionally, varus angles were recorded as negative numbers, while valgus angles were recorded as positive numbers [18] (Fig. 2). At the same time, angles around the knee joint such as mechanical lateral distal femoral angle (LDFA), medial proximal tibial angle (MPTA) and joint line convergence angle (JCLA) were also measured. All measurements were performed independently by two experienced doctors. According to our data and related research, the preoperative valgus angle was divided into three groups (≤ 4°, 4° to 7° and ≥ 7°), and postoperative alignment was also divided into three groups (≤-3°, -3° to 3° and ≥ 3°) [6, 19, 20].

Patients’ clinical outcomes were evaluated based on theirthe American Knee Society (AKS) score [21], and functional outcomes were evaluated based on the Western Ontario and McMaster Universities Arthritis Index (WOMAC) score. To offer more intuitionistic information for statistical analysis, the WOMAC scores were converted into a percentile system, in which higher scores represent better functional outcomes. The scores were obtained through outpatient reviews or follow-up telephone calls and were evaluated by a physician who was blinded to the patient. The Institutional Review Board of Qilu Hospital of Shandong University approved this study (KYLL-202306-066). Informed consent was waived because this wasa retrospective study.

All data were processed by SPSS 27.0 (SPSS, Inc., Chicago, USA). The *t* test was used to compare the data before and after surgery. ANOVA and LSD post hoc tests were used to compare the postoperative score and valgus angle. Multiple linear regression analysis was applied to explore the relationship between imaging parameters and scores. All data satisfy or approximate a normal distribution. A p value less than 0.05 was considered statistically significant.

## Results

The scores of all patients improved after surgery. The scores were related to the valgus condition of the lower limb alignments but were not related to age, sex, BMI, K-L classification, etc.

### Patients’ basic characteristics

There were no significant differences between groups regarding age, sex, BMI and the Kellgren-Lawrence grade (Table 1).

**Table 1:** Comparison of patients’ basic characteristics between different groups *BMI.* Body mass index, *K-L Grade.* Kellgren-Lawrence Grade

*Preoperative valgus*	*≤ 4°*	*4° to 7°*	*≥ 7°*	*Difference*
*N*	*9*	*15*	*27*	
Age	56.89 ± 4.26	60.87 ± 8.53	62.48 ± 8.26	n.s.
BMI	24.74 ± 2.20	26.24 ± 3.70	27.39 ± 4.56	n.s.
Gender (Male/Female)	2/7	4/11	10/17	n.s.
K-L Grade (II/III/IV)	3/6/0	3/11/1	9/12/6	n.s.
*Postoperative valgus*	*≤-3°*	*-3° to 3°*	*≥ 3°*	*Difference*
*N*	*10*	*17*	*24*	
Age	57.80 ± 4.13	62.41 ± 9.29	61.38 ± 8.00	n.s.
BMI	24.90 ± 2.53	27.09 ± 3.24	26.93 ± 4.93	n.s.
Gender (Male/Female)	3/7	5/12	8/16	n.s.
K-L Grade (II/III/IV)	2/7/1	6/8/3	7/14/3	n.s.

*BMI*. Body mass index, *K-L Grade*. Kellgren-Lawrence Grade

### Operation effect

After surgery, there is a significant reduction of the valgus angle (*p* < 0.001), with an average reduction of 5.85 ± 1.35. The LDFA exhibited a significant increase (*p* < 0.001), while the JCLA exhibited a significant decrease (*p* < 0.001). However, there was no significant change in the MPTA. Moreover, the AKS and WOMAC scores exhibited significant improvement after the surgical intervention (*p* < 0.001). (Table 2)

**Table 2:** Comparison of imaging parameters and scores before and after LUKA

	Preoperative	Postoperative	*p* value	Power
Valgus	7.46 ± 3.78	1.62 ± 4.06	< 0.001*	2.66
LDFA	84.19 ± 3.02	86.66 ± 1.93	< 0.001*	2.25
MPTA	88.69 ± 2.21	88.72 ± 1.50	n.s.	1.89
JCLA	2.07 ± 1.26	0.47 ± 1.29	< 0.001*	1.27
AKS	44.51 ± 9.48	90.35 ± 2.95	< 0.001*	10.15
WOMAC	42.48 ± 8.82	90.60 ± 2.72	< 0.001*	9.38

*LDFA.* Lateral distal femoral angle, *MPTA.* Medial proximal tibial angle, *JCLA.* Joint line convergence angle, *AKS.* The American Knee Society (AKS) score, *WOMAC.* Western Ontario and McMaster Universities Arthritis Index

* Indicates a significant difference (*p* < 0.05)

### Valgus alignment

Patients with a preoperative valgus angle greater than 7° had higher postoperative AKS and WOMAC scores than those with a preoperative valgus angle less than 4° (*p* < 0.05). Furthermore, sequential increases in the WOMAC scores were observed in patients with a postoperative valgus angle less than − 3°, between − 3° and 3°, and greater than 3°(*p* < 0.05). Patients with a postoperative valgus angle less than − 3°hada lower AKS score than the rest of the patients (*p* < 0.05). (Table 3)

**Table 3:** Valgus alignment with postoperative scores

*Preoperative valgus*	*N*	*AKS*	*WOMAC*
*≤4°*	*9*	*88.00 ± 3.39*	*87.27 ± 3.16*
4° to 7°	15	90.27 ± 3.39	90.07 ± 2.36
≥7°	27	91.19 ± 2.08	92.01 ± 1.44
*Statistical analysis*		*p value*	
≤ 4° versus 4°to 7°		0.057	0.003*
≤ 4° versus ≥ 7°		0.004 *	< 0.001*
4°to 7° versus ≥ 7°		0.306	0.006*
*Postoperative valgus*	*N*	*AKS*	*WOMAC*
≤-3°	10	86.20 ± 2.82	86.25 ± 1.54
-3° to 3°	17	91.06 ± 1.85	90.50 ± 1.84
≥3°	24	91.58 ± 1.98	92.49 ± 0.92
*Statistical analysis*		*p value*	
≤-3° versus − 3°to 3°		< 0.001*	< 0.001*
≤-3° versus ≥ 3°		< 0.001*	< 0.001*
-3° to 3° versus ≥ 3°		n.s.	< 0.001*

*AKS.* The “Knee Score” part of the American Knee Society (AKS) knee score, *WOMAC.* Western Ontario and McMaster Universities Arthritis Index

Statistical power is 0.41 (preoperative valgus with AKS), 0.70 (preoperative valgus with WOMAC), 0.50(postoperative valgus with AKS) and 0.74 (postoperative valgus with WOMAC) respectively

* Indicates significant difference (*p* < 0.05)

### Linear relationship

We performed a study using multiple linear regression models to evaluate the relationship between imaging parameters and postoperative scores. The results revealed a significant correlation between imaging parameters and the postoperative WOMAC score (adjusted R^2^ = 0.67, *p* < 0.001). We found that for every degree increase in postoperative valgus angle, the WOMAC score increased by 0.59 (*p* < 0.001). Additionally, we also found that for every degree increase in angle change, the WOMAC score decreased by 0.55 (*p* = 0.005). Although there was a correlation between the postoperative AKS score and imaging factors, the impact was mild (adjusted R^2^ = 0.27, *p* = 0.002), and we did not discover any independent influencing factors. (Table 4)

**Table 4:** Multiple linear regression analysis between scores and imaging parameters

	Postop. AKS			Postop. WOMAC		
	*Regression coefficient*	*95% CI*	*p value*	*Regression coefficient*	*95% CI*	*P value*
Postop. valgus	0.27	-0.02 to 0.56	n.s.	0.59	0.41 to 0.78	< 0.001*
Change of valgus	-0.36	-0.96 to 0.24	n.s.	-0.55	-0.92 to -0.18	0.005*
Postop. LDFA	-0.23	-0.69 to 0.23	n.s.	0.19	-0.10 to 0.47	n.s.
Postop. MPTA	-0.27	-0.81 to 0.27	n.s.	0.02	-0.31 to 0.36	n.s.
Postop. JCLA	0.11	-0.70 to 0.92	n.s.	-0.41	-0.91 to 0.09	n.s.
*Total*	*F*	*Adjusted R* ^*2*^	*p value*	*F*	*Adjusted R* ^*2*^	*p value*
	4.64	0.27	0.002*	21.46	0.67	< 0.001*

*LDFA.* Lateral distal femoral angle, *MPTA.* Medial proximal tibial angle, *JCLA.* Joint line convergence angle, *AKS.* The American Knee Society (AKS) score, *WOMAC.* Western Ontario and McMaster Universities Arthritis Index

* Indicates a significant difference (*p* < 0.05)

## Discussion

The most important finding drawn from the study is that patients with postoperative valgus alignment of 3° or more obtained better outcomes after lateral UKA. Moreover, the study revealed that patients with mild preoperative valgus (≤ 4°) generally obtained worse outcomes compared to those with severe valgus (≥ 7°). The study also identified a positive correlation between postoperative valgus angle and functional score, as well as a negative correlation between angle change value and functional score.

Compared to the medial femoral condyle, the lateral femoral condyle has a longer sagittal diameter, is located near the vertical axis, and has a smaller volume. At the same time, the lateral tibial plateau has a smaller posterior slope angle, and the lateral meniscus has a loose connection with the joint capsule [22, 23]. Therefore, the lateral compartment has a greater range of motion, and lateral UKA with a mobile-bearing prosthesis may result in a higher incidence of prosthesis dislocation [10, 11, 24].Many studies have been published to show the relationship between the patient prognosis and their postoperative alignment after medial UKA. Vasso et al. assessed the IKS scores, range of knee motion and postoperative alignment of 125 patients who underwent medial UKA and concluded that patients with postoperative mild varus alignment (4° to 7°) were likely to have good outcomes [6]. More than three thousand medical records were collected in the study by Slaven SE et al., and they found that patients with mild varus alignment of approximately 4° had better postoperative outcomes. Valgus deformity could simulate the progression of lateral compartment osteoarthritis after medial UKA [25]. Compared with medial UKA, lateral UKA is likely to increase the changes in alignment, and the risk of overcorrection is higher in lateral UKA [7]. Van der List, JP et al., using a similar method to investigate the effect of lower limb alignment on lateral UKA, recommended a postoperative valgus angle of 3°-7° [19].

The most common cause of failure of UKA is the progression of OA in the contralateral compartment. Additionally, overcorrection of alignment in UKA may result in a reduction in prosthesis survival time [26–29]. Our investigation has found little research on the influence of lower limb alignment on the outcomes of patients following lateral UKA. In this study, we examined the correlation between postoperative outcomes and lower limb alignment. After surgery, the alignment was classified into three groups: varus (≤ -3°), neutral (-3° to 3°), and valgus (≥ 3°) alignment. We also used a similar classification system to divide the preoperative lower limb alignment into three groups based on 4° and 7°. Lateral UKA is a favourable treatment for KOA with isolated lateral compartment. Patients recovered well after an average of 24months of follow-up, and the alignment in more than half of the patients was corrected to a neutral position (14 of 26 patients). The results show that patients with postoperative varus alignment exhibited significantly worse clinical and functional outcomes than the other two groups. Although valgus patients had higher mean scores than neutral patients, the difference was not significant. Patients with preoperative mild valgus (≤ 4°) alignment obtained worse functional outcomes after lateral UKA. The linear relationship of the preoperative and postoperative lower limb alignment angles illustrated that patients with mild preoperative valgus were more prone to varus deformity postoperatively. In patients with a high risk of varus deformity following lateral UKA, it is recommended to adjust the osteotomy depth or loosen the insertion point of the iliotibial band during the operation to maintain a neutral or mild valgus alignment postoperatively.

This study found that age, sex, and BMI did not have a significant impact on patients’ scores. However, it is important to note that the impacts of these factors on UKA are still a topic of debate and require further research. In a study by Kennedy et al., more than 1000 patients who underwent medial UKA were followed up for a period of ten years. The study results showed that patients older than 75 years had significantly lower OKSs at the end of the ten-year follow-up period [30]. In another study, Ekhtiari et al. found that male patients under the age of 50 had a higher UKA revision rate [31]. In a systematic review and meta-analysis by Salman et al., it was found that, in young patients, there was no significant association between higher revision rates and lower functional scores [32]. In a case‒control study, Polat et al. found that morbid obesity (BMI ≥ 35 kg/m2) is an independent risk factor affecting both functional outcomes and implant survival after UKA [33]. James et al. concluded that obesity should not be a contraindication for medial Oxford UKA. In fact, patients with a BMI of 35 kg/m2 or more benefited the most from this procedure [34]. Giordano et al. found that patients with a BMI greater than 30 kg/m2 were able to recover well after undergoing lateral UKA. This suggests that a BMI over 30 kg/m2 may not be a reliable contraindication for UKA [35]. Because of the small number of lateral UKAs, further research in this area will be needed to support relevant conclusions.

We are aware that our research may have some limitations. The conclusions drawn in this study are limited by the small sample of patients with complete follow-up data. As a result, it is difficult to perform more detailed group analysis for optimal postoperative alignment. The average follow-up period of 27 months may not be sufficient to assess the relationship between implant survival and lower limb alignment, as no surgical failure was observed during this follow-up period.

The study’s results indicate that an optimal postoperative valgus alignment target should be greater than 3°. It is advised to avoid postoperative varus of more than 3°. Additionally, greater attention should be given to patients who present with mild preoperative valgus (≤ 4°) alignment.

## Conclusion

Lateral UKA is an excellent treatment method for lateral compartmental osteoarthritis. Lower limb alignment seems to be an independent predictor of postoperative outcomes. Postoperative valgus alignment (≥ 3°) leads to great clinical and functional outcomes, while varus alignment (≤-3°) is related to undesirable outcomes. Therefore, it is recommended that more than 3° of valgus alignment be maintained after LUKA. Care should be taken during surgery to avoid over-correction, as it may have a negative impact on postoperative functional recovery. In future studies, a larger number of cases must be examined and the follow-up time should be extended to explore the optimal alignment goals and the impact of alignment on implant survival.

### Electronic supplementary material

Below is the link to the electronic supplementary material.

Supplementary Material 1

Supplementary Material 2

Supplementary Material 3

## Data Availability

All data generated during this study are included in supplemental files.
