# Supplementary figures and images for: Effect of lower limb alignment on outcome after lateral unicompartmental knee arthroplasty: a retrospective study
Source: BMC Musculoskelet Disord. 2024 Jan 20;25:82. doi: 10.1186/s12891-024-07208-4 (PMC10799503; doi:10.1186/s12891-024-07208-4)

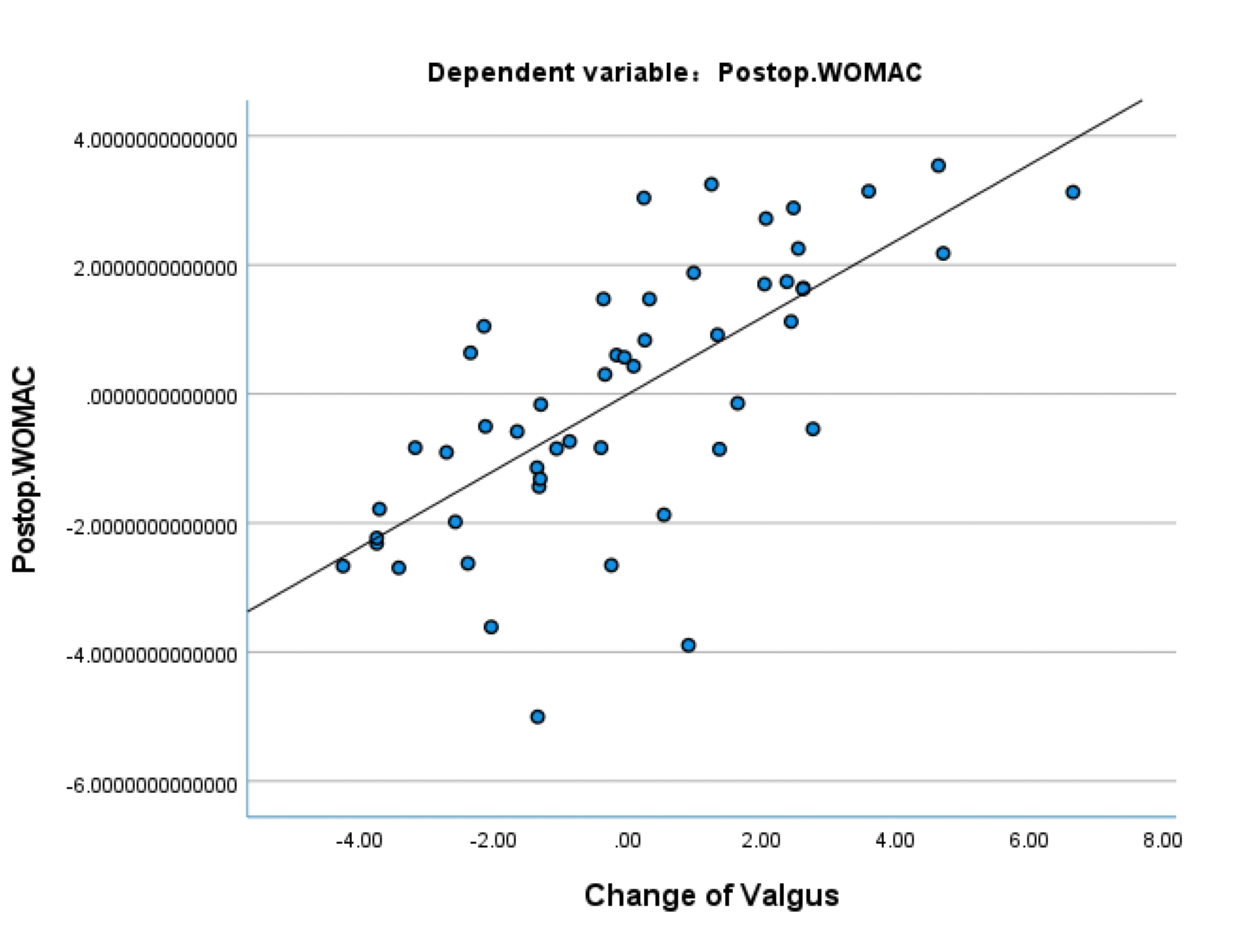

Supplement: Supplementary file 2 — Supplementary Material 2 [file 12891_2024_7208_MOESM2_ESM.png]

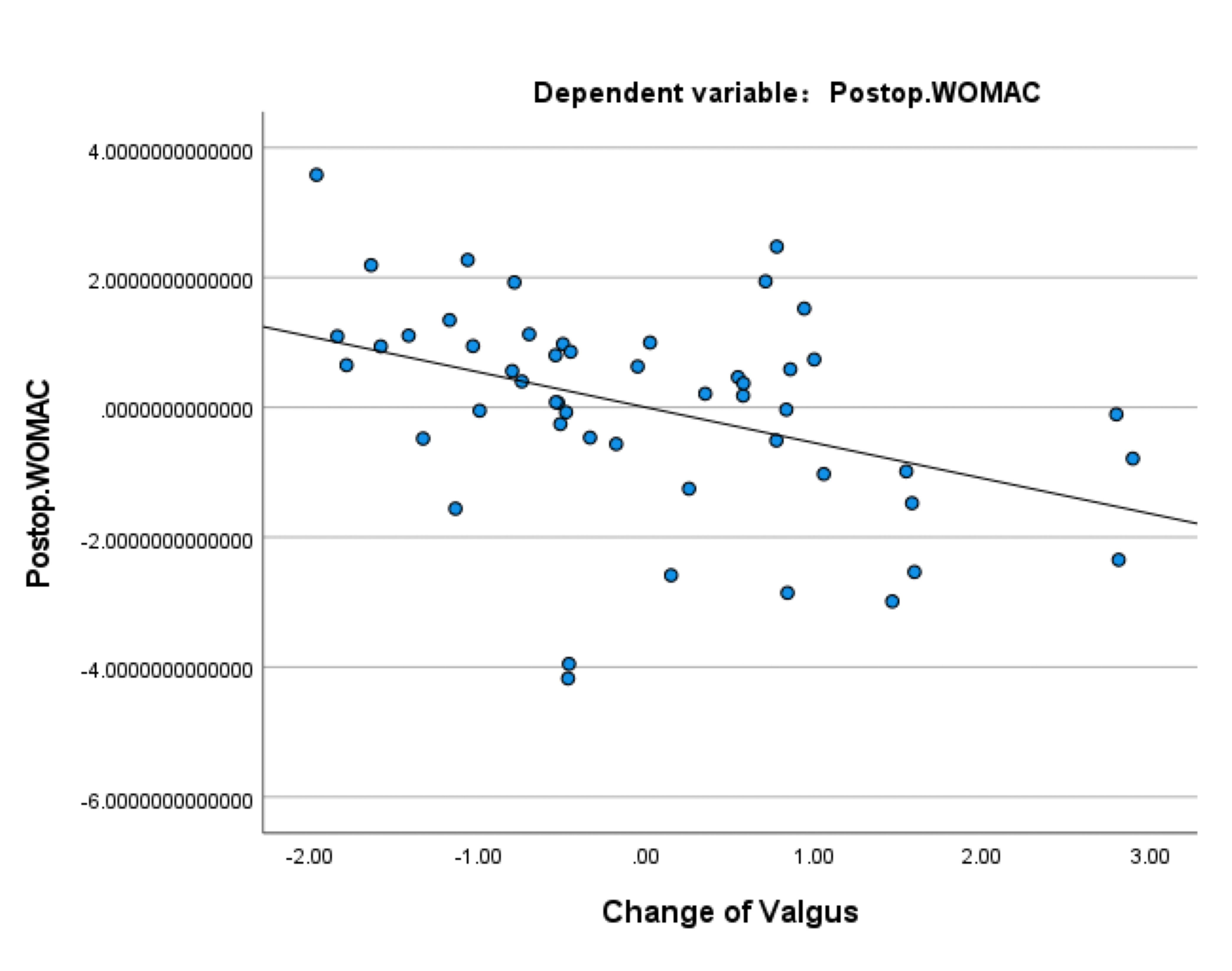

Supplement: Supplementary file 3 — Supplementary Material 3 [file 12891_2024_7208_MOESM3_ESM.png]
